# Methodological Considerations in Evaluating CTRP12 as a Biomarker for In‐Stent Restenosis

**DOI:** 10.1002/clc.70271

**Published:** 2026-02-02

**Authors:** Saleha Khattak, Syed Muhammad Rayyan, Syed Huzaifa Khan

**Affiliations:** ^1^ Khyber Girls Medical College Peshawar Pakistan; ^2^ Ayub Medical College Abbottabad Pakistan; ^3^ Nowshera Medical College Nowshera Pakistan

Dear Editor,

We read with great interest the study by Botao Zhao and Yuchan Shen, which evaluates changes in serum CTRP12 in patients with coronary artery disease (CAD) after the treatment of percutaneous coronary intervention (PCI) and its relationship with in‐stent restenosis (ISR). The authors have addressed an emerging area of vascular biology and present CTRP12 as a possible biomarker with significant prognostic value. Although the findings are intriguing and novel, some aspects of the study must be discussed to appropriately contextualize the conclusions [1].

The selection of patients for the ISR group relied on follow‐up coronary angiography rather than protocol‐mandated, systematic surveillance [2]. As patients who undergo repeat angiography are more likely to be symptomatic or high risk, this raises the possibility that the observed association between CTRP12 levels and ISR may reflect correlations with severity of the disease or symptom burden instead of true anatomical restenosis, potentially overstating the stated predictive ability of the biomarker.

Next, the study does not establish CTRP12 as an independent predictor of ISR. The lack of a multivariable model limits the interpretation of the results as validated predictors of ISR, such as angiographic disease severity (e.g., Gensini score), inflammatory markers like hs‐CRP, and clinical risk factors were not adjusted for [3]. Without demonstrating incremental prognostic value beyond these existing variables, CTRP12 may present as a mediator of underlying inflammation or disease burden rather than an independent biomarker [4].

Moreover, the immediate post‐PCI period is marked by acute vascular injury, endothelial disruption, and inflammatory activation related to balloon inflation and stent deployment. Hence, changes in CTRP12 levels at 24 hours post‐procedure indicate acute procedural trauma rather than mechanisms directly involved in the longer‐term development of ISR [4].

Also, procedural factors such as total stent length, number of stents implanted, lesion complexity, and duration of balloon inflation are known to influence inflammatory responses and ISR risk. Such variables were not accounted for; therefore, lower CTRP12 levels may simply reflect more complex or injurious interventions [5].

Lastly, the single‐center design with a relatively limited sample size restricts external validity. The optimal cut‐off values concluded from a single population may not be generalizable across other demographics. External validation in independent and multicenter cohorts is essential before CTRP12 can be considered for clinical practice.

In summary, while the study contributes valuable data on CTRP12 and its prognostic value, verification bias, absence of a multivariable model, ambiguity related to the timing of biomarker measurement, and possible restricted generalizability hinder the strength of its conclusions. Addressing these limitations in future studies will be crucial to defining the actual utility of CTRP12 clinically in coronary intervention.

## Author Contributions

All authors have read and approved the final manuscript.

## Funding

The authors received no specific funding for this work.

## Ethics Statement

The authors have nothing to report.

## Consent

The authors have nothing to report.

## Conflicts of Interest

The authors declare no conflicts of interest.

## Data Availability

The authors have nothing to report.
